# Efficacy and safety of the orthopaedic manipulation techniques of the Lin School of Lingnan Region in the treatment of adolescent idiopathic scoliosis: protocol of a participant-and-assessor-blinded randomized controlled study

**DOI:** 10.1186/s12891-023-07152-9

**Published:** 2024-01-04

**Authors:** Hing Yu Hung, Wan Ching Kong, Tsz Hei Tam, Ping Chung Leung, Yongping Zheng, Arnold Yu Lok Wong, Zhixiu Lin, Fei Yao, Qiang Tian, Tik Lun Mok, Lyncam Edviano Loo, Kiu Lam Chung

**Affiliations:** 1 School of Chinese Medicine, Faculty of Medicine, The Chinese University of Hong Kong, Shatin, Hong Kong SAR, N.T China; 2 Institute of Chinese Medicine, The Chinese University of Hong Kong, Shatin, Hong Kong SAR, N.T China; 3 Department of Biomedical Engineering, The Hong Kong Polytechnic University, Hung Hom, Kowloon, Hong Kong SAR, China; 4 Department of Rehabilitation Sciences, The Hong Kong Polytechnic University, Hung Hom, Kowloon, Hong Kong SAR, China; 5 School of Acupuncture-Moxibustion and Tuina, Shanghai University of Traditional Chinese Medicine, Shanghai, China; 6 The Second Affiliated Hospital, Guangzhou University of Chinese Medicine, Guangzhou, China

**Keywords:** Scoliosis, Spinal curvature, Spinal diseases, Bone diseases, Musculoskeletal diseases, Manipulation

## Abstract

**Background:**

Adolescent idiopathic scoliosis (AIS) is the most common developmental spine disorder among children. It is characterized by a lateral deviation of the spine that gives rise to the distinctive “S” or “C” shaped bending of the spine. The Lin School of Lingnan Region (LSLR), one of the prominent schools for bare-handed orthopaedic manipulation in southern China, provides preliminary evidences that the orthopaedic manipulation techniques help to correct deviations of the spine. Previous research found that Orthopaedic Manipulation Techniques of LSLR (OMT-LSLR) could reduce the Cobb’s angles in patients with AIS. Therefore, the current study aims to investigate the effectiveness and safety of the OMT-LSLR in treating teenagers with AIS.

**Methods:**

In this participant-and-assessor-blinded randomized controlled clinical trial, 50 participants identified AIS without surgical indications will be recruited and randomized into two groups to receive physiotherapy scoliosis-specific exercises training with either orthopaedic manipulation or sham manipulation treatment for 16 weeks, followed by post-treatment visits at week 24. Primary outcome measure is the change of Scoliosis Research Society-22 (SRS-22) questionnaire score. Secondary outcome measures include Traditional Chinese version of Spinal Appearance Questionnaire (TC-SAQ) score, Italian Spine Youth Quality of Life (ISYQOL) score, the change of Cobb’s angle measured by Xray, and the change of Cobb’s angle, spinal rotation and muscle volume measured by three-dimensional (3D) ultrasound. The trial will be conducted at the Chinese University of Hong Kong Chinese Medicine Specialty Clinic cum Clinical Teaching and Research Centre in Hong Kong (CUHK-CMSCTRC).

**Discussion:**

The results of this study will establish comprehensive clinical evidence about the efficacy and safety of the Orthopaedic Manipulation Techniques of the Lin School of Lingnan Region in the Treatment of Adolescent Idiopathic Scoliosis. One of the characteristics of this trial is that it is a participant-and-assessor-blinded randomized controlled clinical trial with sham manipulation. The study would also apply three-dimensional (3D) ultrasound technology to investigate the relationship between the change of the muscle volume and the spinal curve.

**Trial registration:**

The trial is registered on ClinicalTrials.gov (Identifier: NCT05639023) on December 6, 2022.

## Background

Adolescent idiopathic scoliosis (AIS) is the most common developmental spine disorder among children. It is characterised by a lateral deviation of the spine, with a Cobb’s angle of more than 10 degrees, that gives rise to the distinctive “S” or “C” shaped bending of the spine [1, 2]. According to the World Health Organisation (WHO), it is estimated that 2,000 to 3,000 children aged between 13 and 16 suffer from AIS for every 100,000 adolescents in the world [3]. In China, study found that the overall scoliosis prevalence rate was 5.14% [4] In Hong Kong, the incidence of AIS is reported to be approximately 3.5%, and it is anticipated to increase progressively in the foreseeable future [5]. AIS does not only influence patients’ appearance, but also increases their risk of back pain and sports injuries, as well as affects their normal growth and development [6, 7]. Consequently, these may have a negative impact on their quality of life [8, 9]. For severe cases, the patients might even suffer from chronic pain or impaired cardiopulmonary function [10–12]. At present, the treatment available for AIS include both operative and non-operative options, and they will be prescribed according to the severity of the spinal deformity. As recommended by the Scoliosis Research Society (SRS), operative treatment is indicated for patients with Cobb’s angle greater than 45 degrees [13, 14]. For Cobb’s angle less than 45 degrees, non-operative interventions such as scoliosis bracing and physiotherapy scoliosis-specific exercises (PSSE) are usually considered for patients [15]. Despite having available treatment for AIS, there are still limitations, with the surgical approach having inherent risks and non-operative approaches lacking desirable evidence for the therapeutic outcomes [15–18].

From the perspective of Chinese medicine (CM), AIS is a spinal disorder that can be treated by CM orthopaedic manipulation. Among different orthopaedic manipulation, the Orthopaedic Manipulation Techniques of the Lin School of Lingnan Region (OMT-LSLR) is one of the prominent schools for bare-handed orthopaedic manipulation in southern China [19]. In a clinical case report [20], the combination of Lin School manipulation techniques and exercise interventions showed a decline in Cobb’s angles among patients with AIS. These results may be promising, but they are at most preliminary evidence and further research is definitely warranted for a comprehensive assessment.

This clinical study aims to examine the clinical efficacy and safety of the OMT-LSLR in the treatment of Adolescent Idiopathic Scoliosis. A randomized controlled trial (RCT) with the treatment duration of 16 weeks and post-treatment follow-up period of 8 weeks was carried out. In this study, we will use comprehensive outcome measures such as health-related quality of life assessment and radiology imaging to evaluate the treatment response.

X-ray imaging is the mainstay in AIS diagnosis and to rule out any underlying conditions in the spine [21]. However, patients with AIS may be subject to multiple radiology imaging for curve progression monitoring, which causes high cumulative radiation in their bodies. Exposure to ionizing radiation have been shown to increase the risk of developing cancer according to previous retrospective studies [22]. In this study, EOS x-ray imaging is applicated and each image can reduce approximately16–34 times lesser organ dose as compared with the standard digital radiography [23]. Moreover, imaging before and after manipulation is required for investigating the effect of manipulation on spine. In order to reduce risk form the radiation, 3D ultrasound imaging is used for the continuous assessment. Ultrasound imaging is an alternative assessment in AIS patients [24]. Previous studies have proven that ultrasound images perform very good correlations and agreement compare with Xray measurements of the scoliotic curvature [25]. Also, ultrasound imaging can be used to measure the changes in muscle [26]. Thus, it can provide a detailed profile of the change in spine before and after the manipulation.

While health-related quality of life assessment is a patient reported outcome measures which provides subjective outcome measurements., both X-ray and ultrasound in the study provide objective outcome measurements. This clinical study will be able to provide comprehensive clinical evidence on the efficacy of OMT-LSLR in the treatment of Adolescent Idiopathic Scoliosis.

### Objectives and hypothesis

The primary objective of the study is to evaluate the clinical efficacy and safety of the OMT-LSLR in the treatment of AIS. The secondary objective is to determine the association between the change in the muscle volume and the spinal curve before and after every treatment. The hypothesise is that the OMT-LSLR is effective for the treatment of Adolescent Idiopathic Scoliosis which will balance the musculoskeletal difference between both sides of the patient’s back.

## Methods/design

### Study design

This study is a prospective trial in participants with AIS and is a participant-and-assessor-blinded randomized controlled trial with the treatment duration of 16 weeks and post-treatment follow-up period of 8 week. Fifty eligible participants will be randomly divided into two groups: the manipulation techniques group and the control group. The allocation ratio will be 1:1. participants in the former group will receive both OMT-LSLR and PSSE, whereas those in the latter group will receive sham manipulation technique, which is similar to OMT-LSLR without any therapeutic effect, and PSSE.

### Study setting

This study will be conducted in The Chinese University of Hong Kong Chinese Medicine Specialty Clinic cum Clinical Teaching and Research Centre (CUHK-CMSCTRC) on the CUHK campus, Hong Kong. CUHK-CMSCTRC is a Teaching and Research Clinic in the university which offering Chinese Medicine treatment.

### Study population

Participants with AIS will be recruited from CUHK-CMSCTRC. Advertisements include posters in the Chinese Medicine clinics and promotion on internet platforms, such as Facebook, emails, and website will be made to facilitate community recruitment. Besides, articles will be published in local newspapers and magazines as well as organize health promotion talks to augment the participant recruitment process. Potential participants with AIS who meet the eligibility criteria will be recruited.

### Outcomes

The study will be conducted over a period of 24 weeks with the treatment duration of 16 weeks and post-treatment follow-up period of 8 weeks. The primary outcome measurement is the change of Scoliosis Research Society-22 (SRS-22) questionnaire score at week 16 when the treatment is completed. SRS-22 questionnaire [27] is used to primarily assess the function/activity, pain, self-perceived image, mental health, satisfaction with treatment and other health-related quality of life parameters when the participants finished the manipulation treatment. In this study, the validated Chinese version of SRS-22 [28] will be used to assess the effectiveness of the treatment.

There are multiple secondary outcomes, including the change of SRS-22 questionnaire score at week 8 and 24, the change of Traditional Chinese version of Spinal Appearance Questionnaire score (TC-SAQ) and Italian Spine Youth Quality of Life (ISYQOL) at week 8, 16 and 24. Both TC-SAQ and ISYQOL are questionnaires specifically for AIS patients. The former, TC-SAQ [29] is a questionnaire specifically assess the cosmetic perception and have already been demonstrated to be excellent for the assessment of appearance in patients. The latter, ISYQOL [30] is used to evaluate health-related quality of life for AIS patients. In the study, validated Chinese version of both questionnaires will be used to assess the effectiveness of manipulation treatment during the treatment and post-treatment period [31, 32].

Secondary outcomes also include the change of Cobb’s angle measured by Xray at week 16, and the change of Cobb’s angle, spinal rotation and muscle volume measured by 3D ultrasound imaging before and after every manipulation. The paraspinal muscle volume will be estimated by a customized MATLAB program, which multiplies the cross-sectional area of paraspinal muscles on each image by the thickness of each image and the total number of images of the target paraspinal muscle. The lateral curvature of the spine in the coronal plane measured by Cobb’s method on X-ray radiograph is the gold standard for assessing scoliosis [33], In the study, a standing whole-spine PA radiograph will be used for measurement and evaluated before and after the 24-week study period. If the patient has double curves, the major curve will be calculated for analysis. Moreover, before and after every manipulation, Cobb’s angle, spinal rotation and muscle volume along the spine will be measured from 3D ultrasound imaging captured by Scolioscan Air. Scolioscan Air is a validated radiation-free system for scoliosis assessment by using coronal images of spine generated by a 3D ultrasound volume projection imaging method [34, 35].

### Eligibility

#### Inclusion criteria

Participants must meet all the following criteria for inclusion [36]:

1. Adolescents aged between 10 and 18 who can speak and read Chinese.
2. Fulfil the diagnostic criteria of scoliosis, i.e. Cobb’s angle ≥ 10 degree.
3. Risser grade ranging from 0 to 4.
4. Informed consent agreement signed by both participant and their parents/guardians.
5. Able to participate in follow-up assessments.

#### Exclusion criteria

Participants will be excluded if they meet one of the following criteria [36]:

1. History of spine surgery.
2. Cobb’s angle > 45 degrees.
3. Known to have severe respiratory or cardiovascular comorbidities; vertebral tumours and spinal canal abnormalities; Leukaemia, thrombocytopenia and other bleeding disorders.
4. Known to have cognitive impairment.
5. Documented pregnancy.
6. Wearing scoliosis brace within 1 month.
7. Involved in other interventional clinical studies at the same time.
8. Uncooperative during treatments
9. Being assessed by investigators as unsuitable to participate.

### The assessor and therapist

The participants will be assessed by a registered Chinese Medicine Practitioner with certificate of Scolioscan operation and who is blinded to this study. The manipulation techniques will be performed by a Chinese Medicine Practitioner who is an expert in the Orthopaedic Manipulation Techniques of the Lin School of Lingnan Region with more than 10 years’ experience. The participating subject will receive PSSE training by certified physiotherapists in a physiotherapy centre.

### Interventions

Participants will be received either 12 times orthopaedic manipulation or sham treatment for 16 weeks: The schedule will be three times of treatment per month and less than twice a week. For PSSE, participants are encouraged to perform home exercise every day on their own throughout the study. Participants will receive five sessions of intensive supervised training for first two weeks and then one to two sessions trainings per month for the rest, amounting to the total of 14 sessions supervise training throughout the study.

### The orthopaedic manipulation techniques of the Lin School of Lingnan Region

The Orthopaedic Manipulation Techniques include steps as follow [20]. First, a combination of X-ray examination and palpation will be first conducted to locate the apex of curve before the treatment. Second, the participants will be seated on the treatment chair, with both hands placed behind the neck. The operator will stand at the side of concave curvature, with one arm placed anteriorly to the participant’s neck and the hand holding firm on the participant’s shoulder of the opposite side, while the other hand is fixed at the mid-lower section of the apex of curve. The assistant will firmly anchor the lower limbs. Third, the participant will be requested to bend forward, with the apex of the curve as the pivot point of the spine curvature. After that, forces will be applied using the hand that was placed on the opposite shoulder. This in turn allows the participant’s spine to rotate along with the force applied. Until a resistance is encountered during the rotation, an explosive force will be applied to the spine using the hand. If the participant has double scoliotic curve, the treatment will be implemented first on the inferior site then superior, following the steps as described above.

### Sham manipulation techniques

For sham manipulation techniques, participants will be instructed to sit on a treatment chair after palpation. The operator will stand at the side of concave curvature, with an arm placed anteriorly to the participant’s neck and the hand holding firm on the participant’s shoulder of the opposite side, while the other hand is fixed at the mid-lower section of the apex of curve. The assistant will firmly anchor the lower limbs. However, the participant only need to rotate slowly without an external force applied to the spine. After that, the operator will stand on the other side and repeat the movement. These non-therapeutic manipulations were performed outside the spinal column with adequate joint slack and without soft tissue pre-tension so that any therapeutic force would not apply to the spine. Rotations to both sides are applied to ensure a broad non-specific movement, which would not produce any therapeutic effect.

### Physiotherapy scoliosis-specific exercises

PSSE training will be conducted by certified physiotherapists in a physiotherapy centre. First, a physiotherapist will conduct physical examinations including manual muscle strength test of the back, muscle tension checking and joint range of motion measurements. Then, the physiotherapist will provide exercise training to the participants according to the examination results. The exercises will be performed in supine, side-lying, prone, sitting, standing and other functional positions [37]. Physiotherapist will incorporate breathing exercises and tactile feedback to enhance the recruitment of target muscles (including paraspinal muscles, abdominal muscles, hip and gluteal muscles) in the desired movements and postures.

### Prohibited other treatments

In order to minimize the participants receiving additional AIS treatments during the study, the participants need to agree that they will not seek additional AIS treatment when they sign the informed consent form. The involved therapists and research personnel will routinely remind the participants during each treatment session. If any participants indicate that they receive additional AIS treatments, the research personnel will document it and use it as a co-variate in the subsequent data analysis.

### Safety

No serious or catastrophic adverse events in children treated by manual therapy were reported in the clinical studies or systematic review [38]. For adults, adverse events such as bruising, muscle soreness are short-lived and minor after manual therapy, and most will occur within 24 h and resolve within 72 h. The risk of major adverse events is very low [39, 40].

### Strategies to improve adherence

In order to encourage the participants to complete the study, the entire treatment including all the interventions and the assessments will be provided at no cost. Participants will receive subsidy in return by following the protocol and completing the study. They will be closely in touch with the researchers to report their symptoms.

### Sample size estimation

PASS 12.0 was used for the sample size calculation. The Scoliosis Research Society-22 (SRS-22) questionnaire score was chosen as the primary outcome indicator. The minimal clinically important difference of SRS-22 ranges from 0.4–0.7 [41]. The standard deviation was estimated to be 0.45 from previous studies about non-operative treatments in AIS patients [42]. To detect a clinically meaningful different of 0.4 in SRS-22, with 80% power at the 0.05 significance level and an assumed standard deviation of 0.45, 21 cases in each group are required. Allowing for a dropout rate of 20%, a total of 50 participants will be recruited with 25 participants for each group.

### Randomization and blinding

All eligible AIS participants will be randomly assigned into either the manipulation techniques group and the control group with an allocation ratio of 1:1. A random number table will be generated with a computerized random number generator program. Each computer-generated random number will be matched with either group and then be sorted in ascending order, which causes the list to rearranged sequence.

After the randomization, group name will be displaced by a specific code in order to keep the blinding in the study. The random allocations will be placed in sequentially numbered, opaque, and sealed envelopes. Two sets of the envelope will be prepared, with one set for randomization at the site and another set stored in the investigator’s office for emergency unblinding. Following the initial evaluations, the numbered envelopes will be given to each participant according to the order of each participant admitted to the study.

This process will be performed by a researcher who will not be involved in the participant enrolment, allocation and assessment. There will be no permission for unblinding the outcome assessor or the participants during the study. The participant can only be informed about their group allocation upon completion of the study.

### Participant timeline

**Fig. 1:**
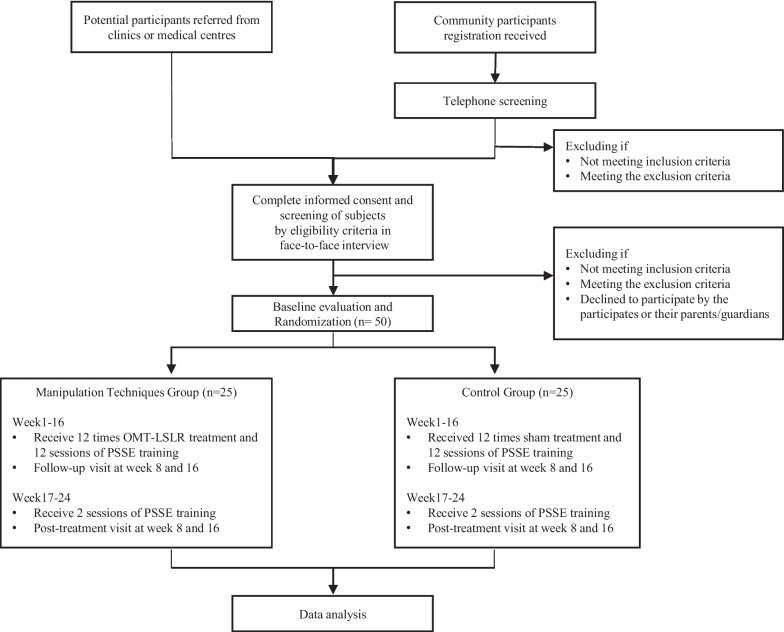
Study flowchart

As shown in the Fig. 1 and Table 1, each participant has a face-to-face interview in screening, a total four visits to the Chinese medicine clinic, 12 times orthopaedic manipulation or sham treatment and 14 sessions of PSSE training throughout the study. Community participants who express interest in our study and agree for our telephone screening will leave their contact to project researcher. Potential participants referred from the clinics or medical centres will be screened directly. In the screening, information about the study will be explained to the participants and their parents/guardians. Both participants and their parents/guardians will then sign informed consent form voluntarily at their agreement. Eligible participants after screening will be arranged a face-to-face interview for eligibility assessment.

**Table 1:**
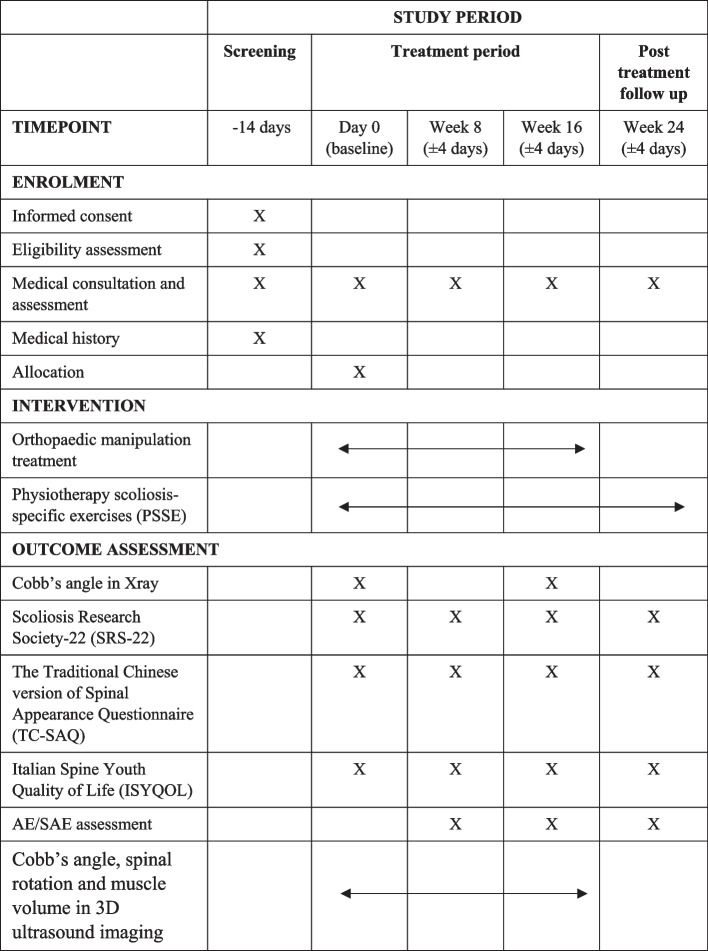
SPIRIT schedule of enrolment, interventions, and assessments

For the baseline visit, eligibility will be checked and participants will take a standing whole-spine PA radiograph for the Cobb’s angle measurement. Then participants will be randomly assigned at a 1:1 ratio to manipulation techniques group or control group. Baseline demographic data and medical history will be taken. SRS-22, TC-SAQ, and ISYQOL will be completed. After that, researcher will schedule the participants for the study treatment and PSSE training.

Participants will return for follow-up at week 8 (± 4 days), week 16 (± 4 days), and be followed by a post-treatment visit at week 24 (± 4 days). SRS-22, TC-SAQ, and ISYQOL will be collected at every follow-up visits. At the 3rd follow-up visit at week 16 a standing whole-spine PA radiograph will be taken. Before and after every manipulation, Cobb’s angle, spinal rotation and muscle volume along the spine will be measured by Scolioscan Air for the 3D ultrasound imaging. Adverse events will be recorded for safety monitoring.

Throughout the study, participants are required to keep a daily record for their compliance for the PSSE and side effects of the study treatment, if any.

### Data management

To protect patient privacy, all research data would be handled in line with Hospital Authority and Hospital’s policy in handling, storage and destruction of participants’ medical records. They would be locked in cabinets where the department keeps the participants’ confidential information. All collected information will be input into a computer with restricted access to investigators only. Only de-identified data will be collected and removed from the premises. All information will be encrypted and only the involved investigators can have access. Password is required to access the data.

### Statistical analysis

All analyses will be conducted according to the intention-to-treat principle. Descriptive statistics will be computed for each of the analysed variables. The primary efficacy analysis will be done by comparing the two groups with respect to score of SRS-22 at baseline and at 8, 16 and 24 weeks after randomization using analysis of covariance (ANCOVA). A complete-case analysis and a linear mixed model analysis of all available data will then be done as supplementary analyses. Repeated measures ANCOVA will be used to test for group differences in the secondary outcomes, including score of TC-SAQ and ISYQOL and the measurement in spinal imaging with adjustment for relevant baseline covariates. Unauthorized treatment will also be added as a covariate in relevant ANCOVA. All statistical tests will be two-sided, and *p* < 0.05 is considered significant difference. The statistical software of SPSS 23.0 will be used for analysis. Adverse events will be categorized, and the percentage of the mild adverse events and serious adverse events will be documented. Chi-square tests will be used to examine differences in the proportion of total and categories of adverse events within each group.

### Data monitoring

This study will be conducted under the supervision of The Joint the Chinese University of Hong Kong–New Territories East Cluster Clinical Research Ethics Review Committee (The Joint CUHK-NTEC CREC). In addition, the Data Monitoring Committee (DMC) has no competing interest in any stages of this study and is completely independent from the sponsor. There will be no interim analysis in this study.

### Participants withdrawal

Participants can withdraw at any time because of personal reasons, adverse events or serious adverse events. An attempt to contact the participant will be made to ask for an explanation for incompliance or termination, and the results of the last assessment should be recorded. For participants who withdraw from the study due to adverse reactions, or treatment failure, appropriate treatment measures should be conducted immediately. Participants are free to withdraw at any time without giving a reason or punishment. All participants are encouraged to attend the follow-up assessment at 24th week for adverse event assessment for the best disease monitoring and participants’ benefit.

### Ethics consideration

This study will be conducted according to the Declaration of Helsinki and International Council for Harmonisation of Technical Requirements for Pharmaceuticals for Human Use (ICH) Guideline for Good Clinical Practice (ICH-GCP). Application for clinical ethical approval will be sought to CUHK-NTEC CREC before the initiation of this study. Participants will be informed that the confidentiality of all information and data will be maintained anonymity. Consent forms should be signed by the participants before the study. All information will be encrypted and only the involved investigators can have access. Password is required to access the data. Participants are free to withdraw at any time without giving a reason or punishment.

### Protocol amendments

In case of protocol modifications, protocol amendments will be submitted CUHK-NTEC CREC for approval (CRE Ref. No. 2022.353).

### Clinical trial insurance

Clinical trial insurance will be purchased according to the University’s policy.

## Discussion

Adolescent idiopathic scoliosis is the most common developmental spine disorder among children. This participant-and-assessor-blinded randomized controlled study allows evaluating the efficacy and safety of an alternative conservative method, the Orthopaedic Manipulation Techniques of the Lin School of Lingnan Region, in the treatment of AIS. Moreover, it helps clarify the effect of OMT-LSLR on the spine and muscle in AIS treatment. If the method does improve the quality of life and reduce the deviations of the spinal curvature in AIS, it can offer an alternative to those patients who cannot or will not opt for surgical treatment. It can also provide a real and meaningful advantage to both the AIS patients and the community.

This study protocol adhered to the Standard Protocol Items: Recommendations for Interventional Trials (SPIRIT) 2013 and SPIRIT-Outcomes 2022 extension [43, 44] in the development and reporting. Being a participant-and-assessor-blinded randomized controlled study, it has several strengths. First of all, quality of evidence is increased by the blinded and randomized study design. Participants and assessors are blinded throughout the study. The blinded design minimizes subjective biases from them. Further, the random allocations sequence preparation is performed by a researcher who are not involved in the participant enrolment, allocation and assessment session. The random allocations sequence generated will be then kept confidential by placing in opaque and sealed envelopes, which minimize the selection bias during the participant recruitment that may affect the outcomes. Second, comprehensive measures are applied to improve adherence from the patients to the study protocol. Standard interventions such as PSSE, and ultrasound assessments are provided for both the manipulation techniques group and the control group. The participants will be closely in touch with the researchers to monitor their symptoms. Nevertheless, participants can receive subsidy in return of following the protocol to complete study. These measures definitely encourage patients to join the study. Third, sham treatment in the control group not only can ensure the blinding but also can increase patient engagement. As the movement of the operator in sham treatment is similar from the real manipulation techniques, sham treatment cannot be easily distinguished by the participants and assessors. Bias can thus be minimized. Additionally, most of the patients would like to have as more interventions as they can. If there is no sham treatment, participant in the control group may think that they have missed some treatment. As a result, they will drop out once they are randomized to control group. Sham treatment can effectively increase the participant compliance as it gives hope to the participants that they still have half chance to receive the manipulation intervention. Forth, 3D ultrasound scanned before and after every manipulation provide a full picture of the change the musculoskeletal situation along the spine. Not only the curvature but also the muscle changes can be monitored to investigate the mechanism of the manipulation techniques.

However, there are some limitations require further comment. First, lack of studies about sham manipulations control groups to verify a standard and unified placebo control group design [45]. Only a few manipulations studies have included a placebo intervention and it is still a challenge for a valid placebo manipulation treatment [46–48]. However, refer to previous study [49], it is generally suggested that the sham manipulations in RCT should be resemble true one in terms of the procedure, treatment frequency and the treatment time to allow similar expectations in the participates from both groups. According to these recommendations, we have designed a set of sham manipulation for OMT-LSLR in AIS treatment with similar movement, except the external force applying on the spine. Further, both treatments will be executed by the same operator to ensure all conditions are as similar as possible. Second, self-compliance for PSSE at home can be an uncertainty which may affect the outcome in the study. Studies demonstrated that PSSE improves health-related quality of life and can halt curve progression or even reduce the Cobb’s angle [50, 51]. The effectiveness of PSSE depends on the compliance of trainings and home exercises [52]. As a result, in order to control the confounding effect, participants need to keep a daily record for their compliance of PSSE throughout the study. Our researchers will keep close contact with the participants and their parents to monitor the compliance of PSSE in both groups.

Based on current studies on conservative treatment of AIS, the quality and quantity of evidence on bracing and PSSE are far ahead of manipulations [53]. The evidence of the effectiveness of OMT conditions remains unproven due to the paucity and low methodological quality of the primary studies [54]. Due to the lack of similar studies for the manipulation in the AIS treatment. This protocol outlines the specific guideline for investigators to study the efficacy and safety of the OMT-LSLR. These guidelines can be applied in other type of manipulation in AIS treatment. It may also help discover the underlying mechanism of manipulation treatment toward the musculoskeletal system. The outcomes have the potential to influence current clinical practice in the conservative treatment in AIS.

## Data Availability

The datasets used and/or analysed during the current study are available from the corresponding author on reasonable request.

## Abbreviations

3D: Three-dimensional
AIS: Adolescent idiopathic scoliosis
ANCOVA: Analysis of covariance
CM: Chinese medicine
CREC: Clinical Research Ethics Review Committee
CUHK-CMSCTRC: The Chinese University of Hong Kong Chinese Medicine Specialty Clinic cum Clinical Teaching and Research Centre
DCM: Data Monitoring Committee
ICH-GCP: International Council on Harmonisation of Technical Requirements for Registration of Pharmaceuticals for Human Use- Good clinical practice
ISYQOL: Italian Spine Youth Quality of Life
OMT-LSLR: Orthopaedic Manipulation Techniques of the Lin School of Lingnan Region
PSSE: Physiotherapy scoliosis-specific exercises
RCT: Randomized controlled trial
SPIRIT: Standard Protocol Items: Recommendations for Interventional Trials
SRS: Scoliosis Research Society
SRS-22: Scoliosis Research Society-22
LSLR: The Lin School of Lingnan Region
TC-SAQ: Traditional Chinese version of Spinal Appearance Questionnaire
